# Optimizing Production in the New Generation of Apricot Cultivars: Self-incompatibility, *S-RNase* Allele Identification, and Incompatibility Group Assignment

**DOI:** 10.3389/fpls.2018.00527

**Published:** 2018-04-27

**Authors:** Sara Herrera, Jorge Lora, José I. Hormaza, Maria Herrero, Javier Rodrigo

**Affiliations:** ^1^Unidad de Hortofruticultura, Centro de Investigación y Tecnología Agroalimentaria de Aragón, Instituto Agroalimentario de Aragón, IA2, CITA, Universidad de Zaragoza, Zaragoza, Spain; ^2^Instituto de Hortofruticultura Subtropical y Mediterránea La Mayora (UMA-CSIC), Málaga, Spain; ^3^Pomology Department, Estación Experimental Aula Dei-CSIC, Zaragoza, Spain

**Keywords:** *Prunus armeniaca*, self-incompatibility, *S*-alleles, *S*-genotype, ovary, pollen tube, pollination, style

## Abstract

Apricot (*Prunus armeniaca* L.) is a species of the Rosaceae that was originated in Central Asia, from where it entered Europe through Armenia. The release of an increasing number of new cultivars from different breeding programs is resulting in an important renewal of plant material worldwide. Although most traditional apricot cultivars in Europe are self-compatible, the use of self-incompatible cultivars as parental genotypes for breeding purposes is leading to the introduction of a number of new cultivars that behave as self-incompatible. As a consequence, there is an increasing need to interplant those new cultivars with cross-compatible cultivars to ensure fruit set in commercial orchards. However, the pollination requirements of many of these new cultivars are unknown. In this work, we analyze the pollination requirements of a group of 92 apricot cultivars, including traditional and newly-released cultivars from different breeding programs and countries. Self-compatibility was established by the observation of pollen tube behavior in self-pollinated flowers under the microscope. Incompatibility relationships between cultivars were established by the identification of *S*-alleles by PCR analysis. The self-(in)compatibility of 68 cultivars and the *S-RNase* genotype of 74 cultivars are reported herein for the first time. Approximately half of the cultivars (47) behaved as self-compatible and the other 45 as self-incompatible. Identification of *S*-alleles in self-incompatible cultivars allowed allocating them in 11 incompatibility groups, six of them reported here for the first time. The determination of pollination requirements and the incompatibility relationships between cultivars is highly valuable for the appropriate selection of apricot cultivars in commercial orchards and of parental genotypes in breeding programs. The approach described can be transferred to other woody perennial crops with similar problems.

## Introduction

Apricot (*Prunus armeniaca* L.) is considered as one of the most delicious temperate fruits (Faust et al., 1998). Apricot is a species of the Rosaceae, one of the most economically important plant families in temperate regions worldwide (Dirlewanger et al., 2004). Although the Latin name of apricot (armeniaca) and later its scientific name (*P. armeniaca*) could wrongly suggest an origin from Armenia, apricot was indeed originated in Central Asia, where the first orchards of apricot were described 406-250 BC (Janick, 2005), whereas Armenia was the route by which apricot first entered Europe. Apricot was already mentioned as Mela armeniaca by Roman authors around 50 A.D., which could indicate its introduction in the Roman empire during the first century (Faust et al., 1998). The English name apricot (apricock in the old spelling) derives from the Arabic and Greek term al-praecox that means “early fruit” (Faust et al., 1998; Janick, 2005). Traditionally, apricot cultivars have been classified in six main groups depending on the geographical origin: Dzhungar-Zailij, East Chinese, European Iranian-Caucasian, Middle-Asian, and North Chinese (Layne et al., 1996).

Apricot cultivars from the Central Asian, the Dzhungar-Zailij, and the Iranian-Caucasian groups are mostly self-incompatible. However, cultivars from the European group, which is the least variable and the most recent, are mainly self-compatible and include most of the commercial cultivars (Mehlenbacher et al., 1991; Hormaza et al., 2007). In the Rosaceae, the incompatibility mechanism to reduce self-fertilization and promote outcrossing is based on cell-cell recognition that is determined genetically by a gametophytic self-incompatibility System (GSI). This mechanism acts through the inhibition of pollen tube growth in the style (de Nettancourt, 2001) and is controlled by a multiallelic locus named *S*, encoding two linked genes that determine the pistil and pollen genotypes (Charlesworth et al., 2005). A ribonuclease (S-RNase), which is a glycoprotein secreted in the style mucilage, determines the allele specificity of the style (Tao et al., 1997) whereas an F-box protein (SFB) specifically expressed in pollen determines pollen allele specificity (Ushijima et al., 2003).

The introduction of an increasing number of new apricot cultivars from different breeding programs is resulting in an important renewal of plant material worldwide (Zhebentyayeva et al., 2012). Thus, the initial classification of six ecogeographical groups is becoming increasingly complex, since many of the new cultivars are derived from crosses between genotypes of different ecogeographical groups (Faust et al., 1998; Halász et al., 2007). Moreover, although most traditional apricot cultivars in Europe are self-compatible (Burgos et al., 1997), the use of self-incompatible cultivars developed in North America as parental genotypes in several breeding programs, with the objective of incorporating resistance to sharka (Hormaza et al., 2007; Zhebentyayeva et al., 2012), is leading to the introduction of new self-incompatible cultivars. As a consequence, there is an increasing need to interplant those new cultivars with cross-compatible cultivars to ensure fruit set in commercial orchards. However, the pollination requirements of many of these new cultivars are unknown.

The pollination requirements of a cultivar can be established by carrying out controlled pollinations in the field and recording the percentage of fruit set. Final fruit set in apricot is usually established during the first 4 weeks following pollination (Rodrigo et al., 2009; Julian et al., 2010). However, incompatibility can be determined more accurately under a fluorescence microscope by the observation of pollen tube growth through the style in self- and cross-pollinated flowers in squash preparations of pistils after staining with aniline blue (Burgos et al., 1993; Rodrigo and Herrero, 1996, 2002; Julian et al., 2010). In self-incompatible genotypes and incompatible crosses, pollen tube growth is arrested in the style and, therefore, fertilization of the ovules is prevented since no pollen tubes reach the ovary. However, in self-compatible genotypes and compatible crosses, pollen tubes can grow along the style and reach the ovary, where fertilization of some of the two ovules can take place. This histochemical approach allows the identification of pollination failure from diverse environmental factors that can affect fruit set under field conditions (Guerra and Rodrigo, 2015).

In addition, advances in the study of the molecular determinants of self-incompatibility have allowed developing tools to analyze the allelic composition of the self-incompatibility locus. Thus, the identification of the *S-RNase* gene in apricot (Romero et al., 2004; Sutherland et al., 2004) allowed developing an *S*-allele genotyping PCR strategy, similar to those developed for cherry or almond (Sutherland et al., 2004). To date, 33 *S*-alleles (*S*_*1*_ to *S*_*20*,_ *S*_*22*_ to *S*_*30*_, *S*_*52*_, *S*_*53*_, *S*_*v*_, and *S*_*x*_), including one allele for self-compatibility (*S*_*c*_), have been identified in apricot (Halász et al., 2005; Vilanova et al., 2005; Zhang et al., 2008; Muñoz-Sanz et al., 2017; Murathan et al., 2017), although additional alleles have been included in the NCBI database and not yet published. These studies allowed the determination of different apricot *S*-genotypes from different countries (Halász et al., 2010; Kodad et al., 2013a,b; Muñoz-Sanz et al., 2017) that are included in, up to now, 17 incompatibility groups (Szabó and Nyéki, 1991; Egea and Burgos, 1996; Halász et al., 2010; Lachkar et al., 2013).

Due to the increasing release of a high number of apricot cultivars in the last years with unknown self-incompatibility genotypes, in this work we analyze the pollination requirements of a group of 92 apricot cultivars, including traditional and new cultivars released from different breeding programs. Self-compatibility was established by the observation of pollen tube behavior under the microscope following self-pollination. Incompatibility relationships between cultivars were established by the identification of *S*-alleles by PCR analysis. The results obtained allowed assigning each cultivar to its corresponding incompatibility group.

## Materials and methods

### Plant material

Leaf and flower samples from 92 apricot cultivars, including traditional cultivars from different origins and new cultivars from different breeding programs (Table 1), were collected from diverse collections for pollination experiments and *S*-*RNase* genotyping.

**Table 1:** Country of origin, number of pistils examined, percentage of pistils with pollen tubes halfway the style, at the base of the style, and reaching the ovule, percentage of style traveled by the longest pollen tube, mean number of pollen tubes at the base of the style, and self-incompatibility (SI) or self-compatibility (SC) of 92 apricot cultivars analyzed in this work.

**Cultivar**	**Country of origin**	**Number of pistils examined**	**Pistils (%) with pollen tubes**			**Percentage of style traveled by the longest pollen tube**	**Mean number of pollen tubes at the base of the style**	**SI/SC**
			**Halfway the style**	**At the base of the style**	**Reaching the ovule**			
AC1	USA	13	100	0	0	82	0	SI
ASF0401	France	17	94	0	0	65	0	SI
ASF0402	France	24	100	0	0	65	0	SI
Avirine (Bergarouge)	France	13	100	0	0	62	0	SI
CA-26 (Almater)	Spain	20	100	5	5	70	0	SI
Colorado	Spain	30	90	0	0	64	0	SI
Cooper Cot	USA	10	100	0	0	65	0	SI
Durobar (Almadulce)	Spain	23	100	0	0	67	0	SI
Farely	France	10	100	0	0	63	0	SI
Feria Cot	France	10	100	0	0	78	0	SI
Flash Cot	USA	10	70	0	0	54	0	SI
Flodea	Spain	11	100	0	0	71	0	SI
Goldbar	USA	20	100	0	0	62	0	SI
Goldrich	USA	72	94	3	3	69	0	SI
Goldstrike 01^a^	USA	40	100	0	0	71	0	SI
Goldstrike 02^a^	USA	20	100	0	0	72	0	SI
Harcot	Canada	44	95	0	0	62	0	SI
Hargrand	Canada	49	100	14	14	77	0	SI
Henderson	USA	47	91	15	9	75	0	SI
Holly Cot	France	20	100	0	0	61	0	SI
JNP	Spain	20	100	5	5	75	0	SI
Lilly Cot	USA	47	96	2	0	67	0	SI
Magic Cot	USA	30	100	0	0	65	0	SI
Maya Cot	France	10	100	0	0	66	0	SI
Medaga	France	10	100	0	0	71	0	SI
Megatea	Spain	10	100	0	0	62	0	SI
Moniqui	Spain	18	100	6	0	79	0	SI
Monster Cot	USA	10	100	0	0	70	0	SI
Muñoz	Spain	21	100	0	0	72	0	SI
Orangered	USA	10	90	0	0	64	0	SI
Pandora	Greece	23	100	4	0	75	0	SI
Peñaflor 01^a^	Spain	29	100	7	7	71	0	SI
Perle Cot	USA	28	93	4	0	72	0	SI
Pinkcot	France	34	97	9	0	83	0	SI
Priabel	France	10	90	10	0	81	0	SI
Robada	USA	25	96	0	0	63	0	SI
Spring Blush	France	40	83	3	3	55	0	SI
Stark Early Orange	USA	51	98	33	16	87	0	SI
Stella	USA	13	100	23	15	85	0	SI
Sun Glo	USA	64	100	2	0	71	0	SI
Sunny Cot	USA	10	100	0	0	65	0	SI
Sweet Cot	USA	20	95	0	0	66	0	SI
Vanilla Cot	USA	20	100	0	0	79	0	SI
Veecot	Canada	29	100	3	3	74	0	SI
Wonder Cot	USA	37	100	0	0	69	0	SI
AC2	USA	10	100	100	100	100	2.2	SC
Aprix 20	Spain	15	100	73	53	100	1.4	SC
Aprix 33	Spain	10	100	100	100	100	1.1	SC
Aprix 9	Spain	14	100	86	64	100	2.1	SC
ASF0404 (Apriqueen)	France	22	100	91	91	100	3	SC
Berdejo	Spain	10	100	100	100	100	1.9	SC
Bergecot	France	20	100	95	95	100	2.5	SC
Canino	Spain	29	100	100	83	100	2.0	SC
Charisma	South Africa	23	100	100	100	100	3	SC
Corbato	Spain	60	100	98	93	100	3.2	SC
Delice Cot	France	15	100	87	53	100	1.1	SC
Faralia	France	11	100	100	100	100	2	SC
Farbaly	France	22	100	86	77	100	2.0	SC
Farbela	France	6	100	100	100	100	1.8	SC
Farclo	France	9	100	100	89	100	1.4	SC
Fardao	France	9	100	100	100	100	4.1	SC
Farfia	France	10	100	100	100	100	3	SC
Farhial	France	10	100	100	100	100	3	SC
Farius	France	12	100	100	100	100	2	SC
Farlis	France	22	100	100	100	100	2	SC
Fartoli	France	10	100	100	100	100	3	SC
Flopria	Spain	10	100	100	100	100	2	SC
Golden Sweet	USA	21	100	95	95	100	2	SC
Lady Cot	France	26	100	77	77	100	2.3	SC
Lorna	USA	17	100	100	100	100	3	SC
Luizet	France	10	100	90	80	100	1	SC
Medflo	France	8	100	100	100	100	1.9	SC
Mediabel	France	12	100	100	100	100	1.2	SC
Mediva	France	9	100	89	89	100	2.3	SC
Mirlo Anaranjado	Spain	10	100	100	100	100	2.1	SC
Mirlo Blanco	Spain	10	100	100	100	100	2	SC
Mitger	Spain	50	100	100	100	100	2.4	SC
Palsteyn	South Africa	30	100	100	100	100	3	SC
Paviot	France	12	100	91	91	100	1.2	SC
Peñaflor 02^a^	Spain	6	100	83.3	66.6	100	1.4	SC
Pepito del Rubio	Spain	12	100	100	90	100	2.2	SC
Playa Cot	France	10	100	100	70	100	1.7	SC
Pricia	France	9	100	100	100	100	2	SC
Primidi	France	9	89	78	78	100	2	SC
Rouge Cot	France	10	100	90	70	100	1.55	SC
Rubista	France	19	95	89	89	100	1.7	SC
Sandy Cot	France	10	100	100	100	100	2.3	SC
Soledane	France	21	100	100	100	100	3	SC
Swired	Switzerland	9	100	100	90	100	1.8	SC
Tadeo	Spain	36	100	97	97	100	2.4	SC
Tom Cot	USA	10	100	100	100	100	3	SC
Victor 1		14	100	93	93	100	2.1	SC

a *Diverse origin*.

### Pollination experiments

To explore self-(in)compatibility, self-pollinations of the 92 apricot cultivars were carried out in the laboratory. Pollen tube growth was observed in self-pollinated flowers under the microscope (Table 1). As control, a group of flowers of each cultivar were cross-pollinated with pollen from “Canino” or “Katy,” which are considered as universal pollinizers for apricot (Zuriaga et al., 2013).

Pollen was extracted from flowers collected at the balloon stage. For this purpose, the anthers were removed and dried at laboratory temperature during 24 h. After that, pollen grains were sieved by using a fine mesh (0.26 mm) and used immediately or frozen at −20°C until further use. Pistils were obtained from flowers collected 1 day before anthesis, at balloon stage. After the removing of petals, sepals and stamens, the pistils were maintained on wet florist foam at laboratory temperature (Rodrigo and Herrero, 1996). For each self- and cross-pollination, a group of 20–25 flowers were hand pollinated with the help of a paintbrush 24 h after emasculation. After 72 h, they were fixed in ethanol (95%)/acetic acid (3:1, v/v) during 24 h, and conserved at 4°C in 75% ethanol (Williams et al., 1999). When observations of pollen tube growth were not clear, each cross was repeated every year during the flowering period up to 4 years. In order to evaluate pollen viability, after hand pollination, pollen from each cultivar was scattered on a solidified pollen germination medium (Hormaza et al., 1996). After 24 h, preparations were observed under the microscope. Pollen grains were considered viable when the length of the growing pollen tubes was higher than the pollen grain diameter.

For histochemical preparations, the pistils were washed three times for 1 h with distilled water and left in 5% sodium sulphite at 4°C for 24 h. Then, to soften the tissues, they were autoclaved at 1 kg/cm^2^ during 10 min in sodium sulphite (Jefferies and Belcher, 1974). To stain callose, the softened pistils, were stained with 0.1% (v/v) aniline blue in 0.1 N K_3_PO_4_ (Linskens and Esser, 1957). The observation of pollen tube behavior along the style was performed by a Leica DM2500 microscope (Cambridge, UK) with UV epifluorescence using 340–380 bandpass and 425 longpass filters. The percentage of style traveled by the longest pollen tube and the mean number of pollen tubes at the base of the style were recorded on at least 10 pistils in each cross. Cultivars were considered as self-incompatible when pollen tube growth was arrested in the style in most pistils from self-pollinated flowers, and as self-compatible when more than half of the pistils displayed at least one pollen tube reaching the base of the style.

### DNA extraction

For the identification of *S-*alleles, young leaves were collected in spring. Genomic DNA from 92 cultivars (Table 2) was isolated following the protocol described by Hormaza (2002) and using a DNeasy Plant Mini Kit (Qiagen, Hilden, Germany). NanoDrop™ ND-1000 spectrophotometer (Bio-Science, Budapest, Hungary) was used to measure DNA concentrations to analyze the quantity and quality of DNA.

**Table 2:** Incompatibility group (I.G.) and *S-RNase* genotype of 92 apricot cultivars analyzed in this study and 30 additional cultivars analyzed in previous studies.

**I.G**.	***S*-RNase genotype**	**Cultivars analyzed in this study**	**Cultivars analyzed in previous studies**	**References**
I	*S_*1*_S_*2*_*	AC1^x^		
			Hargrand	2, 5, 14
			Katy	13, 14
			Goldrich	1, 2, 3, 5, 6, 10, 13, 14
			Castleton	14
			Farmingdale	9
			Giovanniello	9
			Lambertin-1	2, 14
II	*S_*8*_S_*9*_*	Pinkcot^x^		
		Perle Cot^x^		
			Ceglédi óriás	5, 8
			Cologlu	12
			Kadioglu	12
			Ligeti óriás	5, 8
			Seftalioglu	12
			Szegedi M.	14
III	*S_*2*_S_*6*_*	ASF0401^x^		
		Avirine (Bergarouge)^x^		
		Moniqui		2, 6, 14
			Iri Bitirgen	12
V	*S_*2*_S_*8*_*	Holly Cot^x^		
		Sweet Cot^x^		
			Alyanak	12
			Ziraat Okulu	12
VIII	*S_*6*_S_*9*_*	Orangered^w^		14
		ASF0402^x^		
		Wonder Cot^x^		
		Stark Early Orange^w^		14
		Feria Cot^x^		
		Sunny Cot^x^		
		JNP^x^		
			Cataloglu	12
			Ozal	12
			Soganci	12
XVIII^z^	*S_1_S_*3*_*	Cooper Cot^x^		
			Perfection	1
XIX^z^	*S_*2*_S_*3*_*	Mayacot^x^		
		Sun Glo		2, 3, 4, 6
XX^z^	*S_*2*_S_*9*_*	Magic Cot^x^		
		Goldstrike 02^v^^,^^x^		
			Hasanbey	12
XXI^z^	*S_*3*_S_*8*_*	Spring Blush^x^		
		Lilly Cot^x^		
			Kayseri P.A	12
XXII^z^	*S_*3*_S_*9*_*	Durobar (Almadulce)^x^		
		Henderson^w^		14
		Flodea^x^		
			Akcadag Günay	12
XXIII^z^	*S_*7*_S_*9*_*	Goldbar^x^		
			Kurukabuk	12
	*S_*2*_*	Pandora^x^		
		Veecot		
		Muñoz^x^		
		Peñaflor 01^v^^,^ ^x^		
		Hardgrand		
		Goldrich		
			Búlida	9
			Lorna^y^	9
			Perla	9
	*S_*3*_*	Colorado^y^		
			Ninfa	9
	*S_*4*_*	Harcot		
	*S_*6*_*	Stella		
	*S_*8*_*	Vanilla Cot^x^		
		Robada^x^		
			Katy	7
			Krimskyi Medunec	5
	*S_*9*_*	Flash Cot^x^		
		Goldstrike 01^v^^,^^x^		
		CA-26 (Almater) ^x^		
		Farely^x^		
		Medaga^x^		
		Megatea^x^		
		Monster Cot^x^		
		Priabel^x^		
Self-compatible cultivars	*S_*2*_S_*c*_*	Berdejo^x^		
		Canino		3, 10, 13
		Paviot^x^		
		Pepito del Rubio		2, 3
		Peñaflor 02^v^^,^^x^		
		Bergecot^x^		
		Mediva^x^		
		Primidi^x^		
		Sandy Cot^x^		
	*S_*3*_S_*c*_*	Pricia^x^		
		Rubista^x^		
	*S_*6*_S_*c*_*	Aprix 20^x^		
		Aprix 9^x^		
		Faralia^x^		
		Farlis^x^		
		Medflo^x^		
		Mediabel^x^		
	*S_*7*_S_*c*_*	Charisma^x^		
	*S_*9*_S_*c*_*	AC2^x^		
		Flopria^x^		
		Tom Cot^x^		
	*S_*c*_*	Soledane^x^		
		ASF0404 (Apriqueen)^x^		
		Mirlo Blanco		11
		Mitger		
		Tadeo		
		Corbato^y^		
		Aprix 33^x^		
		Delice Cot^x^		
		Farbaly^x^		
		Farbela^x^		
		Farclo^x^		
		Fardao^x^		
		Farfia^x^		
		Farhial^x^		
		Farius^x^		
		Fartoli^x^		
		Lady Cot^x^		
		Mirlo Anaranjado		
		Luizet^x^		
		Playa Cot^x^		
		Swired^x^		
		Rouge Cot^x^		
	*S_1_S_2_*	Lorna^x^^,^^y^		
		Palsteyn^x^^,^^y^		
	*S_*2*_S_*9*_*	Victor 1^x^		
	*S_3_*	Golden Sweet^x^		

*(1) Egea and Burgos (1996); (2) Burgos et al. (1998); (3) Alburquerque et al. (2002); (4) Sutherland et al. (2004); (5) Halász et al. (2005); (6) Vilanova et al. (2005); (7) Chen et al. (2006); (8) Halász et al. (2007); (9) Donoso et al. (2009); (10) Raz et al. (2009); (11) Egea et al. (2010); (12) Halász et al. (2010); (13) Zuriaga et al. (2013); (14) Muñoz-Sanz et al. (2017)*.

v *Diverse origin*.

w *S_9_ and S_17_ have been considered the same allele*.

x *S-RNase genotypes first reported in this study*.

y *Cultivars in which S-RNase genotype reported herein differs from that reported in other studies*.

z *Incompatibility groups first reported in this study*.

### *S-RNase* allele identification by PCR analysis

Amplification reactions for the first intron region of the *S-RNase* gene were carried out with the combination of the fluorescently labeled forward primer SRc-F (5′-CTCGCTTTCCTTGTTCTTGC-3′) with the reverse primer SRc-R (5′-GGCCATTGTTGCACAAATTG-3′; Romero et al., 2004; Vilanova et al., 2005). PCR amplifications were carried out in 15 μl reaction volumes, containing 10x NH_4_ Reaction Buffer, 25 mM Cl_2_Mg, 2.5 mM of each dNTP, 10 μM of each primer, 100 ng of genomic DNA and 0.5 U of BioTaq™ DNA polymerase (Bioline, London, UK). The temperature profile used had an initial step of 3 min at 94°C, 35 cycles of 1 min at 94°C, 1 min at 55°C and 3 min at 72°C, and a final step of 5 min at 72°C.

The sizes of the products obtained by PCR were analyzed in a CEQ™ 8000 capillary electrophoresis DNA analysis system (Beckman Coulter, Fullerton, CA, USA) and compared and classified according to Vilanova et al. (2005) and Kodad et al. (2013b). Primers Pru-C2 (5′-CTTTGGCCAAGTAATTATTCAAACC-3′) and Pru-C4R (5′-GGATGTGGTACGATTGAAGCG-3′) were used for the amplification of the second intron region as recommended by Vilanova et al. (2005), but with the addition of 10 cycles and using 55°C of annealing temperature as indicated by Sonneveld et al. (2003). Amplified fragments of the second intron were separated on 1% (w/v) agarose gels and DNA bands were visualized using the nucleic acid stain SYBR Green (Thermo).

### Sequencing of genomic PCR products

Two PCR fragments of 420 and 430 bp obtained by the automatic sequencer were isolated using the NucleoSpin Gel and PCR Clean-up (Macherey-Nagel). Cloning was performed using CloneJET PCR Cloning Kit (Thermo) and by electroporation in *E. coli* Single-Use JM109 Competent Cells (Promega). The search for similarities in the sequences of the NCBI database was performed with BLAST (http://www.ncbi.nlm.nih.gov/BLAST, version 2.2.10). The 420 bp fragment resulted in a fragment of 414 bp after one sequencing reaction whereas the initial 430 bp fragment resulted in a fragment of 421 bp after two sequencing reactions.

## Results

### Pollination experiments

**Figure 1:**
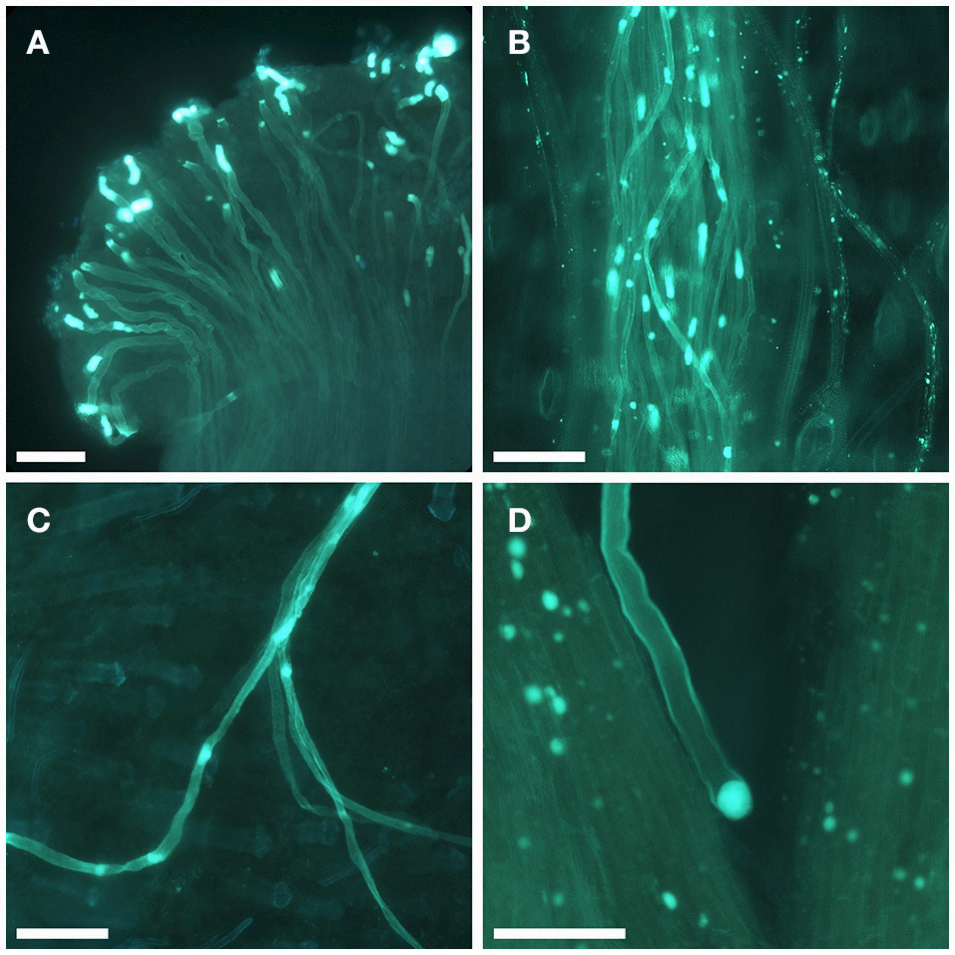
Pollen tube growth in self-pollinated apricot flowers. **(A)** Pollen grains germinating at the stigma surface. **(B)** Pollen tubes growing along the style. **(C)** Pollen tubes reaching the base of the style. **(D)** Pollen tube arrested in the style. Scale bars, 100 μm.

Self-compatibility of 92 apricot cultivars was established by the observation of pollen tube behavior in pistils under the microscope after self-pollinations (Table 1). Germinated pollen grains were observed in the stigma (Figure 1A) in all the pollinations performed. The establishment of self-incompatibility or self-compatibility could be carried out for all the cultivars. Approximately half of the cultivars (47) behaved as self-compatible, displaying most pistils with pollen tubes growing along the style (Figure 1B) and at least one pollen tube reaching the base of the style (Figure 1C). On the other hand, in 45 cultivars pollen tubes arrested their growth in the style (Figure 1D) and no pollen tubes reached the base of the style in most of the pistils. Consequently, these cultivars were considered as self-incompatible. As expected, in all cross-pollinations pistils displayed pollen tubes at the base of the style. Between one and four pollen tubes at the base of the style were observed in self-compatible cultivars.

### *S-RNase* allele PCR analysis

**Figure 2:**
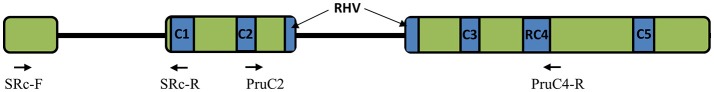
Gene structure of the *P. armeniaca RNase* gene. Genomic sequence of the *S*_*4*_ allele showing the exons in green square, the primers used for the identification of *S*-alleles and the five conserved regions (C1, C2, C2, C3, RC4, and C5), and one hypervariable region (RHV) in blue square.

To confirm the results obtained in the pollination experiments, PCR analyses using specific primers from conserved regions of the apricot *S-RNase* locus were used to identify the *S-RNase* alleles of 92 apricot cultivars (Table 2). The information reported herein has been compiled with the *S-RNase* genotype of 30 additional cultivars previously determined showing the compatibility relationships among all the cultivars whose *S-RNase* genotype is known (Table 2). Cultivars have been allocated according their *S-RNase* alleles in 11 incompatibility groups, six of them reported here for the first time. Some cultivars with previously reported *S*-genotypes were initially used to confirm the size of *S-RNase* alleles previously identified using the primer pairs SRc-F and SRc-R (Vilanova et al., 2005) that amplify the first intron of the apricot *S*-*RNase* and allowed identifying the *S-RNase* alleles of the rest of the cultivars analyzed (Figure 2).

Two alleles, *S*_*1*_ and *S*_*7*_, could not be distinguished with the primers SRc-F/SRc-R, since these alleles showed similar fragment sizes in the first intron (Table 3). Thus, the PruC2/PruC4R primer combination designed from *P. avium S-RNase*-cDNA sequences (Tao et al., 1999) was additionally used to amplify the second intron. The alleles *S*_*c*_ and *S*_*8*_ had also similar fragment sizes in the first intron, and, in this case, the self-compatibility or self-incompatibility observed in the pollination experiments was used to distinguish between both alleles in each cultivar. A fragment of 420 or 430 bp was detected in some of the cultivars. These band sizes are close to the *S*_*6*_ allele, which has been reported as 424 bp (Kodad et al., 2013b) or 423 bp (Halász et al., 2010). To elucidate if the 430 bp and 420 bp bands obtained by an automatic sequencer correspond to new or pre-existing alleles, both fragments were cloned and sequenced, resulting in fragments of 421 and 414 bp, respectively.

**Table 3:** *S*-alleles identified or/and sequenced in *Prunus armeniaca*.

**Alleles**	**Gen bank accession**	**Sequence**	**Fragment 1st intron**	**Fragment 2nd intron**	**References**
*S_*c*_*	EF491872/DQ386735	Partial CDS, 1st intron	355^a^^,^^e^	2800^c^	Halász et al., 2007
*S_*1*_*	AY587561	CDS, 1st and 2nd intron	408^a^^,^^e^	2260^b^^,^^e^	Romero et al., 2004
*S_*2*_*	AY587562	CDS, 1st and 2nd intron	334^a^^,^^e^	990^b^	Romero et al., 2004
*S_*3*_*			274^a^^,^^e^	~450^b^^,^^e^	Vilanova et al., 2005
*S_*4*_*	AY587564	CDS, 1st and 2nd intron	249^a^^,^^e^	247^b^^,^^e^	Romero et al., 2004
*S_*5*_*			375^a^	1400^b^	Vilanova et al., 2005
*S_*6*_/S_*52*_*	KF951503 (*S_*52*_*)	CDS, 1st and 2nd intron	421^a^^,^^e^	1386^b^^,^^e^	Unpublished
*S_*7*_*			408^a^^,^^e^	900^b^^,^^e^	Vilanova et al., 2005
*S_*8*_*	AY884212	Partial CDS, 2nd Intron	355^a^^,^^e^	691^b^	Feng et al., 2006; Halász et al., 2007
*S_*9*_*	AY864826	Partial CDS	414^a^^,^^e^	749^b^^,^^e^	Feng et al., 2006
*S_*10*_*	AY846872	Partial CDS, 2nd Intron		583^d^	Feng et al., 2006
*S_*11*_*	DQ868316	Partial CDS, 2nd Intron		464^d^	Zhang et al., 2008
*S_*12*_*	DQ870628	Partial CDS, 2nd Intron		360^d^	Zhang et al., 2008
*S_*13*_*	DQ870629	Partial CDS, 2nd Intron		401^d^	Zhang et al., 2008
*S_*14*_*	DQ870630	Partial CDS, 2nd Intron		492^d^	Zhang et al., 2008
*S_*15*_*	DQ870631	Partial CDS, 2nd Intron		469^d^	Zhang et al., 2008
*S_*16*_*	DQ870632	Partial CDS, 2nd Intron		481^d^	Zhang et al., 2008
*S_*17*_*	DQ870633	Partial CDS, 2nd Intron		487^d^	Zhang et al., 2008
*S_*18*_*	DQ870634	Partial CDS, 2nd Intron		1337^d^	Zhang et al., 2008
*S_*19*_*	EF133689	Partial CDS, 2nd Intron		546^d^	Zhang et al., 2008
*S_*20*_*	EF160078	Partial CDS, 2nd Intron		1934^d^	Zhang et al., 2008
*S_*22*_*	HM053569	Partial CDS, 2nd Intron		550^d^	Unpublished
*S_*23*_*	EU037262	Partial CDS, 2nd Intron		505^d^	Wu et al., 2009
*S_*24*_*	EU037263	Partial CDS, 2nd Intron		168^d^	Wu et al., 2009
*S_*25*_*	EU037264	Partial CDS, 2nd Intron		583^d^	Wu et al., 2009
*S_*26*_*	EU037265	Partial CDS, 2nd Intron		289^d^	Wu et al., 2009
*S_*27*_*	EU836683	Partial CDS, 2nd Intron		230^d^	Wu et al., 2009
*S_*28*_*	EU836684	Partial CDS, 2nd Intron		948^d^	Wu et al., 2009
*S_*29*_*	EF185300	Partial CDS, 2nd Intron		285^d^	Wu et al., 2009
*S_*30*_*	EF185301	Partial CDS, 2nd Intron		956^d^	Wu et al., 2009

a *Amplified using SRc-(F/R)*.

b *Amplified using Pru-C2 and Pru-C4R*.

c *Amplified using Pru-C2 and Pru-C3R*.

d *Amplified using other primers*.

e *Our results*.

The 421 bp fragment showed a 99% identity with the *S*_*52*_ present in the NCBI database. This allele was initially included in the NCBI database and unpublished but, recently, it has been reported in some Turkish apricot cultivars (Murathan et al., 2017). Since the *S*_*6*_ allele had not been previously sequenced, the *S*_*52*_ allele could indeed correspond to the *S*_*6*_ allele. The *S*_*6*_ allele could also be identified with the primers Pru-C2/Pru-C4, showing a PCR-fragment of around 1400 bp (1386 bp) that included the second intron; a 1386 bp fragment was also amplified in the *S*_*52*_ allele with the same primer combination strongly suggesting that *S*_*6*_ and *S*_*52*_ could be the same allele. Thus, in this work, the 421 bp fragment was assigned to the *S*_*6*_ allele.

The sequence of the 414 bp fragment showed high sequence similarity to *S*-alleles from other *Prunus* species, but not to any *S*-allele of *Prunus armeniaca* present in NCBI databases. The second intron of this allele was amplified with the primers Pru-C2/Pru-C4, showing a PCR-fragment of around 700 bp. Its cloning, sequencing and alignment revealed a 99% identity with the *S*_*9*_ allele [AY853594 (Feng et al., 2006)] and, consequently, the 414 bp fragment was assigned to the *S*_*9*_ allele.

The 45 self-incompatible cultivars were grouped in incompatibility groups according to their *S* genotypes following the numbering proposed by Halász et al. (2010) and Lachkar et al. (2013). While 26 of the cultivars analyzed were assigned to 11 different incompatibility groups, 19 cultivars were not assigned since only one *S*-*RNase* allele was detected (Table 2). *S*_*2*_ was the most frequent allele, appearing in 22 cultivars, followed by *S*_*c*_ (21), *S*_*9*_ (19), *S*_*6*_ (16), *S*_*3*_ (10), *S*_*8*_ (6), and *S*_*1*_ (4), while *S*_*7*_ was the least frequent allele found in only two cultivars.

## Discussion

Self-pollination of the 92 apricot cultivars analyzed in this work and observation of pollen tubes under the microscope showed that 47 behaved as self-compatible and 45 as self-incompatible. The self-(in)compatibility of 68 cultivars is reported herein for the first time. The results in the remaining 23 cultivars have been compared with previous reports in which self-(in)compatibility was determined by the evaluation of the percentage of fruit set after self-pollinations in the field (Egea and Burgos, 1996; Rodrigo and Herrero, 1996; Burgos et al., 1997; Egea et al., 2010; Muñoz-Sanz et al., 2017) or by the observation of pollen tube growth in pistils after self- and cross-pollinations (Egea and Burgos, 1996; Rodrigo and Herrero, 1996; Egea et al., 2010; Milatovic et al., 2013a,b). Thus, results herein agree with previous reports for “Canino,” “Corbato,” “Luizet,” “Mirlo Blanco,” “Mirlo Anaranjado,” “Mitger,” “Palsteyn,” “Paviot,” “Pepito del Rubio,” “Tadeo,” and “Tom Cot” as self-compatible, and also for “Bergarouge,” “Goldrich,” “Goldstrike,” “Harcot,” “Hargrand,” “Moniqui,” “Orangered,” “Pinkcot,” “Robada,” “Stark Early Orange,” “Stella,” “Sun Glo,” and “Veecot” as self-incompatible.

Approximately half of the cultivars analyzed (49%) were self-incompatible, a very high percentage compared to the situation some years ago when most European cultivars were self-compatible (Mehlenbacher et al., 1991), including the most traditional cultivars (Burgos et al., 1997). The other half (51%) were self-compatible. Due to this dramatic increase in the number of self-incompatible apricot cultivars, knowing their pollination requirements is necessary in order to choose compatible pollinizers in designing new commercial orchards as well as in selecting parental genotypes in apricot breeding programs.

The first case of cross-incompatibility of apricot cultivars in the European group was reported in the early 1990s in the Spanish cultivar Moniqui (Egea et al., 1991). The first incompatibility group in apricot, which consisted of three North American cultivars, was established several years later based on microscopic observations (Egea and Burgos, 1996), and, later, a more extensive study with 123 apricot cultivars, reported self-incompatibility in 42 cultivars (Burgos et al., 1997). Afterward the S-RNase proteins of seven *S*-alleles (*S*_*1*_–*S*_*7*_) and one allele associated with self-compatibility (*S*_*c*_) were identified; the proteins were separated by non-equilibrium pH gradient electrofocusing (NEpHGE) in a gel that was later stained for ribonuclease activity (Alburquerque et al., 2002). The identification of the gene involved in GSI allowed *S*-allele identification by PCR analysis (Romero et al., 2004). In this first study, three *S*-alleles were sequenced (*S*_*1*_, *S*_*2*_, and *S*_*4*_) (Romero et al., 2004). In a later study, *S*-allele genotyping using the SRc-F and SRc-R primers, which have also been used in our study and amplify the first intron, identified four self-incompatibility alleles (*S*_*3*_*, S*_5_*, S*_6_, and *S*_*7*_) and one allele for self-compatibility (*S*_*c*_) (Vilanova et al., 2005). Nine additional *S*-alleles (*S*_*8*_–*S*_*16*_) were identified by Halász et al. (2005) in 23 apricot accessions, mostly from Hungary. These last 13 alleles (*S*_*3*_, *S*_*5*_–*S*_*16*_) were only identified by PCR analysis. From then on, several studies have identified additional *S*-alleles by sequencing [*S*_*9*_ and *S*_*10*_ (Feng et al., 2006); *S*_*11*_–*S*_*20*_ (Zhang et al., 2008); *S*_*23*_–*S*_*30*_ (Wu et al., 2009)], but some of them have only been included in the NCBI database and not yet published. Some unpublished alleles such as *S*_*52*_ (Murathan et al., 2017)/*S*_*6*_ (our results) and *S*_*22*_ (Muñoz-Sanz et al., 2017), have already been associated to several cultivars. Most of these studies have been performed in apricot cultivars from different origins like China, Turkey, Hungary, or Spain and, in some cases, the use of different primers or just the inaccuracy of band identification in a gel or even analyzed by an automatic fragment analyzing system can result in misidentification and the appearance of numerous homologies (Muñoz-Sanz et al., 2017). For example, differences in reported fragment size can be found in the *S*_*2*_ [334 bp herein; 327 bp in Vilanova et al. (2005); 332 bp in Kodad et al. (2013b)] or *S*_*c*_ [358 bp herein; 353 bp in Vilanova et al. (2005); 355 bp in Kodad et al. (2013b)] alleles in addition to the *S*_*6*_ allele mentioned above. Moreover, many of the *S*-alleles, such as *S*_*10*_–*S*_*30*_ alleles, in which the first intron is still unknown, have only been partially sequenced.

From the 92 cultivars analyzed herein, 74 have been reported for the first time. Two alleles could be identified in most cultivars. However, a single allele was identified in 19 self-incompatible cultivars that may be due to inefficient PCR amplification of the *S*-*RNase* allele, in which the PCR primers may have a preferential amplification of the detected allele or caused by mismatching of PCR primers. Alternatively, the similar size of two alleles that show overlapping PCR fragments could make their identification difficult. Thus, the identification of additional *S*-*RNase* alleles in these genotypes requires more work focused on characterizing the *S*-locus. In 22 self-compatible cultivars, the identification of a unique allele could also be due to homozygosis of the self-compatible allele (*S*_*c*_). Higher *S-*allele frequencies in the cultivars analyzed correspond to alleles *S*_*2*_, *S*_*c*_, and *S*_*9*_ (19–22%). The allele *S*_*c*_ is associated with self-compatibility, and the alleles *S*_*2*_ and *S*_*9*_ are present in different cultivars resistant to sharka from USA (“Goldrich,” “Henderson,” “Orangered,” and “Sun Glo”) and Canada (“Hargrand” and “Veecot”), which have been used as parental genotypes in different breeding programs (Hormaza et al., 2007; Zhebentyayeva et al., 2012). On the other hand, the allele *S*_*7*_ is the least frequent and is present in only two cultivars: “Charisma” from South Africa, and “Goldbar” from USA.

Results herein, while confirmed the *S-RNase* genotype of 16 cultivars reported in previous studies, showed differences in the *S-RNase* genotype of “Corbato,” “Colorado,” “Lorna,” and “Palsteyn” reported previously (Burgos et al., 1998; Alburquerque et al., 2002; Vilanova et al., 2005; Donoso et al., 2009; Raz et al., 2009; Muñoz-Sanz et al., 2017). To clarify their *S*-genotype, it would be necessary to identify their *S*-alleles in additional samples of the same cultivars. In this sense, sequencing can reveal the differences between a band fragment of an initial size, such as in our case, in which the initial fragment of 420 and 430 bp resulted in a *S-*allele of 414 bp (*S*_*9*_) and 421 bp (*S*_*6*_) respectively, after sequencing. Moreover, the sequence of the *S*_*6*_ (fragment of 421 bp) could reveal its similarity to *S*_*52*_ that has been recently assigned to Turkish apricot cultivars (Murathan et al., 2017).

The self-incompatibility identification has allowed allocating the cultivars to their corresponding incompatibility groups. This information, compiled with those reported in previous studies, has allowed describing six new incompatibility groups (XVIII–XXIII). Thus, self-incompatible cultivars within the same incompatibility group have the same *S*-genotype and are genetically incompatible with each other. On the other hand, cultivars with at least one different *S-*allele are placed in different incompatibility groups and are inter-compatible.

Although self-incompatibility was observed in nearly half of the cultivars analyzed herein, self-compatibility was still found in a good number of apricot cultivars. Self-compatibility has been related to particular *S*-alleles in different self-incompatible *Prunus* species, as almond (Fernández i Martí et al., 2014; Company et al., 2015), Japanese apricot (Ushijima et al., 2003), peach (Tao et al., 2006; Hanada et al., 2014), sour cherry (*Prunus cerasus* L.) (Yamane et al., 2003), and sweet cherry (Wunsch and Hormaza, 2004; Marchese et al., 2007; Cachi and Wünsch, 2014). The breakdown of the incompatibility system has been reported in these *Prunus* species affecting the function of *S*-locus in the stylar *S*-determinant (*S*-*RNase*) and in the pollen *S*-determinant (F-box protein, SFB). It has also been related to mutations outside the *S*-locus (Hegedus et al., 2012; Company et al., 2015). In apricot, self-compatibility has been related with the insertion of a 358-bp fragment in the *S*-haplotype-specific F-box (*SFB*) gene. This insertion has been reported in self-compatible Spanish (Vilanova et al., 2006) and Hungarian (Halász et al., 2007) cultivars and, since they only differ in two nucleotides in the intron region, a common origin for them has been suggested (Halász et al., 2007). Moreover, a detailed study on the *S*_*8*_ allele, a very common allele in Hungarian cultivars, suggests that *S*_*c*_ could derive from the *S*_*8*_ allele (Halász et al., 2007). Thus, the insertion of 358-bp in the *SFB* gene is only present in the *S*_*c*_ haplotype and both alleles can only be distinguished by primers designed based on the *SFB* sequence such as the degenerate primers AprSFB-F1/R (Halász et al., 2007) or, as in our case, by pollination experiments.

The microscopy observations herein revealed self-compatibility in “Lorna” and “Palsteyn,” cultivars with the *S*_*1*_*S*_*2*_ genotype, “Victor 1” with the genotype *S*_*2*_*S*_*9*_, and “Golden Sweet” with the allele *S*_*3*_, but the *S*_*c*_ allele was not reported. A different mutation that is not linked to the *S*-locus has also been related to self-compatibility with a loss of pollen *S*-activity (Vilanova et al., 2006) and it has recently been associated with the *M*-locus (Zuriaga et al., 2013; Muñoz-Sanz et al., 2017). Due to its limited distribution, it has been suggested that the *M*-locus would be in a very early stage of dispersion (Muñoz-Sanz et al., 2017). Interestingly, the *S*_*c*_ allele is generally found in cultivars with the *m*-haplotype suggesting that this could be the result of a relaxed constrain for self-compatible selection (Muñoz-Sanz et al., 2017). A similar *S*-genotype is also found in self-compatible “Katy,” in which the compatibility was associated to the *M*-locus (Zuriaga et al., 2013). A further analysis could reveal if self-compatibility in “Lorna,” “Palsteyn,” “Victor 1,” and “Golden Sweet” is also related to the *M*-locus.

Studies on *S*-allele identification can also provide information on the genetic diversity of the species. Thus, Chinese cultivars are mostly self-incompatible with a higher number of *S*-alleles (Zhang et al., 2008) compared with the limited number of *S*-alleles found in Western countries (Halász et al., 2007). A study on genetic diversity using AFLP markers showed a decreasing genetic diversity of apricot cultivars from the former USSR to Southern Europe (Hagen et al., 2002; Halász et al., 2007), which is coherent with the Asian origin of the species. However, although most traditional European cultivars are self-compatible (Burgos et al., 1997), due to breeding and crosses with Asian cultivars, the number of self-incompatibility cultivars is recently increasing in Western countries (Muñoz-Sanz et al., 2017; Murathan et al., 2017) and our study confirms this trend. Moreover, our results also show six new incompatibility groups in addition to the initial 17 incompatibility groups described so far.

Thus, the results obtained in this work using pollen tube growth observation in self-pollinated pistils, has allowed establishing the self-compatibility or self-incompatibility of 92 cultivars, including traditional and the main current cultivars as well as new cultivars from different breeding programs. *S*-*RNase* allele identification has allowed the allocation of a number of cultivars to their corresponding incompatibility groups, determining the incompatibility relationships between cultivars. The combination of these two complementary approaches results in valuable information for the appropriate selection of cultivars in commercial orchards and for the selection of parental genotypes in apricot breeding programs and a similar approach could be used in other woody perennial crops.

## Author contributions

JR, JH, and MH: conceived the study; SH, JL, JR, JH, and MH: designed the experiments and wrote the paper; SH and JL performed the microscope observations, the PCR analysis, and analyzed the data; SH and JL contributed equally to this work.

### Conflict of interest statement

The authors declare that the research was conducted in the absence of any commercial or financial relationships that could be construed as a potential conflict of interest.
